# Enrichment of Fruit Peels’ Nutritional Value by Solid-State Fermentation with *Aspergillus ibericus* and *Rhizopus oryzae*

**DOI:** 10.3390/molecules29153563

**Published:** 2024-07-28

**Authors:** Liliana P. Araújo, Helena Vilela, Joana Solinho, Rita Pinheiro, Isabel Belo, Marlene Lopes

**Affiliations:** 1 CEB–Centre of Biological Engineering, Campus de Gualtar, University of Minho, 4710-057 Braga, Portugal; lparaujo@ceb.uminho.pt (L.P.A.); ibelo@deb.uminho.pt (I.B.); 2 Center for Research and Development in Agrifood Systems and Sustainability, Polytechnic Institute of Viana do Castelo, Avenida Atlântico, 4900-348 Viana do Castelo, Portugal; 3 LABBELS–Associate Laboratory, Braga/Guimarães, 4710-057 Braga, Portugal

**Keywords:** banana peels, orange peels, Plackett–Burman, Box–Behnken, total protein, antioxidant activity

## Abstract

The fruit processing industry is responsible for disposing of huge amounts of byproducts, especially fruit peels (FPs), which are often discarded in landfills. Using FPs in biotechnological processes contributes to a circular economy, reducing the environmental burden of FPs and increasing the revenue of the fruit processing industry. This study was focused on upgrading the nutritional value of orange (OPs) and banana (BPs) peels by solid-state fermentation (SSF) with filamentous fungi. SSF factors (moisture, fermentation time, inoculum size, ammonium sulfate (AS), and corn steep liquor (CSL)) and fungi species (*Aspergillus ibericus* and *Rhizopus oryzae*) were studied by a variable screening Plackett–Burman design. Both fungi grew on untreated FPs, increasing their protein content and antioxidant activity. Moisture, AS, and CSL were further studied by a Box–Behnken design with *A. ibericus*. Fermented OPs at 70% moisture and 0.005 g/g AS increased their protein content by 200%, whereas BPs at 70% moisture and 0.005 g/g CSL increased by 123%. Fermented peels were enriched in protein, fiber, and minerals, with a low content of carbohydrates and soluble sugars. Fermented OPs and BPs showed higher antioxidant activity than unfermented peels. The SSF of these FPs is an innovative approach that contributes to obtaining rich nutrient-fermented peels for food.

## 1. Introduction

Peels and seeds comprise around one-third of fruits, and after consumption or industrial processing of fruits, these byproducts are frequently utilized for animal feed, incinerated, or disposed of in landfills, at a cost to the manufacturer [1,2]. Banana (BPs) and orange (OPs) peels, in particular, are high-nutrient byproducts that are abundantly generated and can be acquired at a low cost. In 2019, approximately 22% of harvested oranges produced annually in the world (76.3 million metric tons) were processed [3], generating a huge amount of peels. BP accounts for about 40% of the total weight of fresh bananas and approximately 36 million metric tons of BPs are generated during industrial processing (e.g., ice cream, yogurt, baby food, bread, cakes, chips, etc.) and human consumption of bananas [4,5,6]. These fruit peels are rich in carbohydrates, dietary fiber, lipids, and minerals (e.g., calcium and potassium) but have a low protein content [4,7,8,9,10]. Currently, OPs and BPs are usually disposed of in landfills or used for agricultural fertilization. Despite the potential to generate high-value co-products from banana and orange cultivation, only a small portion of the total volume of OPs and BPs has been utilized, mainly as dietary fiber-rich food ingredients (e.g., flour, candied peels, bread, cakes, etc.), as animal feed, or in the formulation of beauty products [1,2,11].

Although the potential of fruit peels’ nutritional value improvement by solid-state fermentation (SSF) with filamentous fungi for food and feed applications has been demonstrated [12,13,14], this is still a relatively unexplored field. SSF has gathered a lot of attention within biotech industries owing to its great potential to produce a wide range of added-value compounds, including feedstock or ingredients for food, chemical, pharmaceutical, or biofuel industries. SSF is carried out with minimal free water and uses a solid substrate, usually agricultural residues or food industry byproducts, that functions as a physical support and nutrient source for the growth and metabolic activity of the microorganisms [15,16,17]. SSF potentially mimics the natural growth conditions of several microbial species, the concentrated environment often leads to higher yields of desired products, and the low moisture content reduces the likelihood of contamination by unwanted microorganisms [15,18]. Furthermore, SSF presents a simpler process design, with reduced operational costs and minimal waste generation, making it more economical and environmentally friendly [16,18,19]. Filamentous fungi are especially suitable for SSF, as they have the capability of growing in several substrates [9,20] and are remarkably known for being capable of penetrating and growing on solid substrates with low water activity, thus enabling them to benefit from the advantages associated with SSF [9,20,21,22].

The use of fruit peels as a substrate in SSF can be an effective strategy to obtain valuable bioactive compounds while addressing the ever-growing consumption demands of society, promoting byproduct valorization, reducing environmental pollution, and promoting circular economy practices. Therefore, this work intends to contribute to a sustainable economy by upgrading the nutritional value of banana and orange peels by SSF. There are few studies employing these fruit peels as a substrate in fungal SSF for nutritional enrichment [23,24]. This study explored the application of SSF using *Aspergillus ibericus* and *Rhizopus oryzae* to enhance the nutritional value of OP and BP, while evaluating the potential of these fermented fruit peels for human consumption, considering their composition in protein, fiber, carbohydrates, lipids, etc. Firstly, a Plackett–Burman experimental design was employed for a fast screening of the factors (moisture content, fermentation time, inoculum size, ammonium sulfate concentration, corn steep liquor concentration, and fungi species) affecting the total protein content, reducing sugars concentration, and antioxidant activity of fermented OPs and BPs. A Box–Behnken design was then performed in an attempt to optimize the levels of moisture, ammonium sulfate, and corn steep liquor which maximize the protein content of OPs and BPs fermented by *A. ibericus* for 7 days. The fermented OPs and BPs with the highest protein content were characterized and evaluated for their nutritional value for food or feed applications.

## 2. Results and Discussion

### 2.1. Composition of Orange and Banana Peels

The SSF efficiency can be affected by the chemical composition of solid substrates, including nutrient availability, moisture content, and the presence of inhibitory compounds, which can influence microbial growth and enzyme activity. For instance, high cellulose and hemicellulose concentrations may induce the production of lignocellulolytic enzymes, while the protein content can promote the synthesis of proteolytic enzymes such as protease [25]. The OPs and BPs were characterized for initial moisture, carbohydrates, soluble reducing sugars, crude fiber, pectin, total protein, total lipids, ashes, total soluble phenols, and antioxidant activity (Table 1).

In general, both fruit peels had a low content of total protein, though BPs had 1.5-fold higher protein than OPs. OPs had a higher content of carbohydrates, reducing sugars, pectin, and total phenols than BPs. By contrast, BPs were richer in crude fiber and total lipids and had a higher content in water. The antioxidant activity was similar in both fruit peels’ extracts. The chemical composition of fruit peels depends on factors such as the fruit variety, growing conditions, and ripeness. The moisture content of OPs used in this study is in the range of values found in the literature, which varied from 62% to 86% [10,26]. The carbohydrate concentration of OPs is in line with the values reported by other authors (53–86.3%) [27,28,29,30,31]. The values of crude fiber, pectin, crude protein, and lipids of OPs fall in the range of those found in the literature. Mahmoud et al. [26] quantified 10% of crude fiber and Twinomuhwezi et al. [32] obtained 16% of pectin. Approximately 7% of crude protein and 2% of lipids were reported by Rivas et al. [33]. The reducing sugars obtained in this work were slightly lower than those attained by Carranza-Méndez et al. [10]. The antioxidant activity of OP extracts was similar to that reported by Özcan et al. [34], but the OPs used by the authors had a considerably lower concentration of total phenols.

BPs had a moisture content slightly higher than that reported by Chau and Huang—88% [35]. The carbohydrate concentration was lower than that found in the literature (43–46%) [31,36]. The crude fiber falls in the range of values of other works (11–26%), though this factor seems to vary considerably between BPs used [31,36,37]. The reducing sugars in BPs largely depends on the species variety and ripeness of the fruit. The concentration obtained in this study was significantly higher than others found in the literature, ranging from 0.09% to 5% [38,39]. Mahardiani et al. [40] reported a pectin content of 10–12% in BPs, which is significantly higher than the value quantified in this study. BP analyzed by Eshak [36] had a similar content of crude protein (8.7%) and total lipids (4.5%) to those of BPs used herein. Based on the literature, the BPs in this study had a lower total phenols content and antioxidant activity compared to the work of Ibiyinka et al. [41].

BPs had a higher ash content (Table 1) and were richer in minerals (Table 2) than OPs. The ash content of OPs is similar to that reported by Carranza-Méndez et al. [10] and Garcia-Amezquita et al. [42]. Similarly, the ash content of BPs is in the range of values found by Emaga et al. [43] and Segura-Badilla et al. [44].

The composition of minerals found in fruit peels can be influenced by plant species and environmental conditions. Regarding the macromineral composition of OPs, the main components were calcium and potassium, while sodium was present in lower quantities. This composition aligns with the one reported by Czech et al. [45]. On the other hand, concerning microminerals, strontium was the main constituent, whereas nickel was detected in small amounts. The macrominerals found in BP were rich in potassium with low amounts of calcium, magnesium, and phosphorous. Manganese emerged as the predominant micromineral, followed by strontium, whereas nickel was present in residual quantities. Aboul-Enein et al. [7] reported a micromineral composition of 93.6 mg/g of potassium, 4.4 mg/g of calcium, 7.1 mg/g of magnesium, and 0.9 mg/g of phosphorus in BP.

### 2.2. Plackett–Burman Design

The effect of several factors on the nutritional value of OPs and BPs fermented by filamentous fungi under SSF was evaluated using a Plackett–Burman experimental design. Table 3 shows the composition of the final fermented peels regarding their total protein content, soluble reducing sugars, and antioxidant activity.

Untreated OPs and BPs demonstrated the potential to be used as substrates by *A. ibericus* and *R. oryzae* species, increasing their nutritional value. The content of total protein ranged from 5.44% (run 12) to 13.33% (run 7) after the SSF of OPs and from 7.74% (run 12) to 11.01% (run 4) after the SSF of BPs. In general, protein content increased in all experiments comparatively to unfermented peels, especially in OP experiments. Particularly in runs 7 (OP) and 4 (BP), protein content increased by 145% and 39%, respectively. Experiments 7 and 11, which have in common fungus *A. ibericus*, yielded the highest protein content in fermented OPs. This suggests that this fungal species has a strong capacity to grow in these peels, given the correlation between high protein content and biomass concentration [46].

In general, an increase in the antioxidant activity of fermented peels was observed comparatively to unfermented peels. Particularly in experiments 9 (OP) and 12 (BP), the antioxidant activity of fermented peels increased 55% and 252%, respectively. It has been demonstrated that SSF offers a promising approach to enhancing the antioxidant potential of byproducts. Filipe et al. (2020) observed an increase in the antioxidant activity of a mixture of the olive mill and winery byproduct extracts after SSF with *A. ibericus* [47]. Likewise, Sousa et al. [48] demonstrated that SSF with *R. oryzae*, *A. ibericus*, and *A. niger* significantly enhanced the antioxidant activity of oilseed cake extracts.

The content of reducing sugars varied greatly depending on the experimental conditions. Fungal species excrete hydrolytic enzymes that hydrolyze complex carbohydrates of fruit peels into sugar monomers [49], which can be used for biomass and metabolite production [48,50]. Fibers of fruit peels can also be hydrolyzed by fungal enzymes, releasing soluble sugars [51]. As a result, and depending on the conditions and fungus strain, soluble sugar content may increase, as proven by Zamora et al. [52]. The authors demonstrated that temperature, substrate concentration, and fermentation time had a significant effect on converting artichoke byproducts into reducing sugars by Pleurotus ostreatus PLO6.

**Figure 1:**
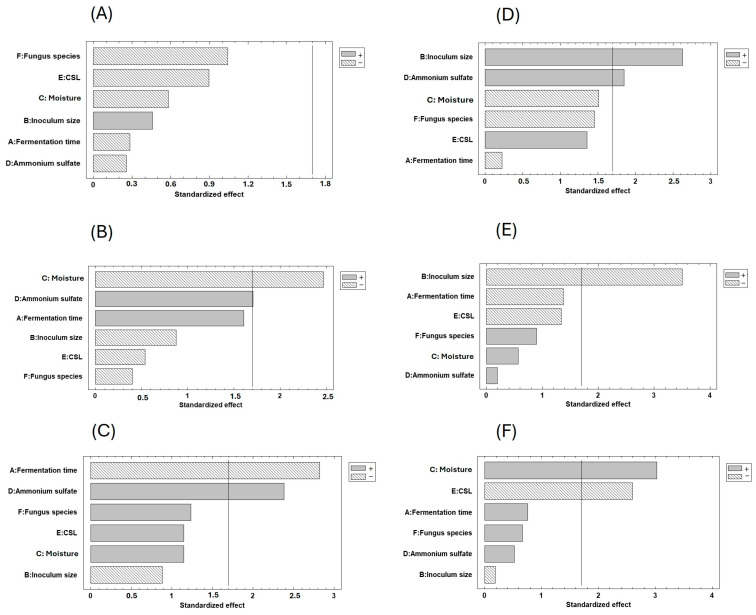
Pareto chart illustrating the standardized effects of several solid-state fermentation independent variables (time, inoculum size, initial moisture, ammonium sulfate concentration (AS), corn steep liquor concentration (CSL), and fungal species) on total protein (**A**,**D**), reducing sugars (**B**,**E**), and antioxidant activity (**C**,**F**) of orange peels (left column) and banana peels (right column).

The effect of the independent variables on the content of total protein, reducing sugars, and antioxidant activity of fermented fruit peels was represented by the standardized effects diagram’s Pareto chart (Figure 1). Standardized effects that surpassed the vertical line were deemed statistically significant if their value was greater than the t-limit (1.7) line.

In the range of values tested for each independent variable, none of them had a significant effect on the total protein content of OPs (Figure 1A). Yet, fungus species (*A. ibericus*), 60% moisture, and non-addition of CSL were the factors that had the highest effect on the total protein content. The inoculum size (5 × 10^7^ spores/g) and ammonium sulfate concentration (0.01 g/g) significantly affected the total protein content on fermented BP. The moisture content, fungus species, and CSL concentration had similar statistical effects and fermentation time was the variable with the lowest influence (Figure 1D). Many works have reported an increase in the total protein content of fermented substrates. For instance, the protein of the brewer’s spent grain [53], distiller’s dried grains with solubles [54], and plant feedstuff mixture [55] increased after SSF with *A. niger* or *A. ibericus*. Several fungal species (*A. niger*, *A. uvarum*, and *A. ibericus*) were screened for protein production in olive, brewery, and wine byproducts, being the highest protein content obtained in SSF with *A. ibericus* [56]. By contrast, the second component of a principal component analysis separated *A. ibericus* from *A. niger* by a lower protein content in the SSF of plant feedstuff mixture [55].

The fermentation time (7 dsays) and ammonium sulfate supplementation had the lowest effect on total protein. In a multi-strain SSF with *A. niger*, *C. utilis*, and *B. subtilis* (at a ratio of 1:1:2), the highest protein content of Moringa oleifera leaf meal was obtained with a total inoculum size of 24%, at 32 °C for 6.5 days and with an initial moisture content of 60% [57]. In the SSF of rapeseed or sunflower meals with *Rhizopus oryzae*, protein content was significantly affected by the relative moisture and temperature, with the highest protein production achieved at low moisture and high temperature. Furthermore, a moisture content exceeding 60% hindered fungal growth due to limited gas exchange, imposing anaerobic conditions on the culture [58]. Olorunnisola et al. [59] observed that the supplementation with high concentrations of KH_2_PO_4_, KCl, NH_4_H_2_PO_4_, and MgSO_4_·7H_2_O improved the protein content of BPs 4-fold through SSF with *Phanerochaete chrysosporium* and *C. utilis*. The consumption of ammonium ions might activate growth-dependent products and prompt hyphae development and spore formation. Furthermore, SO4^2−^ may cause mycelia branching and stimulate the secretion of enzymes and co-factors.

The moisture content (60%) and supplementation with 0.01 g/g ammonium sulfate had a significant positive effect on reducing sugars’ concentration in the fermented OPs (Figure 1B). Though its effect was not statistically significant at a confidence level of 95%, a fermentation time of 14 days positively affected the content of reducing sugars. The inoculum size, CSL concentration, and fungus species had the lowest effect on reducing sugars’ concentration. The inoculum size (1 × 10^7^ spores/g) was the only variable with a statistical effect on reducing sugars’ concentration in fermented BPs (Figure 1E). The fermentation time and CSL concentration had similar statistical effects, and ammonium sulfate concentration was the variable that least affected the reducing sugars’ concentration. Perceiving the kinetic profiles of reducing sugars’ generation and consumption during SSF is challenging because the fungal species consume sugars to grow and produce metabolites, including enzymes that hydrolyze the fibers and lignocellulosic fraction of fruit peels to more free sugars, which can be once more consumed. Regardless of the conditions (inoculum size, temperature, and fermentation time) tested, the concentration of reducing sugars in fermented *Moringa oleifera* leaf meal was significantly lower than that of the unfermented one since *C. utilis* utilized sugar monomers to proliferate, avoiding the suppressive effects of metabolites [57]. A reduction in the reducing sugars’ concentration was observed after the SSF of brewers’ spent grain, exhausted olive pomace, and vine-shoot trimmings [56]. By contrast, no significant differences were observed in the free sugar content of plant feedstuff mixing [55] and exhausted grape mark [56] after SSF with filamentous fungi.

The supplementation with 0.01 g/g ammonium sulfate and a fermentation time of 7 days were statistically significant for the antioxidant activity of fermented OPs (Figure 1C). The effect of the other variables was statistically similar. The antioxidant activity of fermented BPs was positively influenced by the high moisture content (75%) in the absence of CSL, and the inoculum size had the lowest effect (Figure 1F). The antioxidant activity is usually related to the phenolic compounds of byproducts and depends on the substrate composition, SSF conditions, and microbial species. Most phenolic compounds are found in a conjugated form, attached to lipids, sugars, amines, and organic acids, reducing their antioxidant potential. During SSF, enzymatic hydrolysis by carbohydrate-degrading enzymes produced by fungi can release these phenolic compounds, thereby enhancing their antioxidant activity [60]. The works found in the literature do not provide consistent results as some studies indicate an improvement in antioxidant activity, while others report a decrease. For instance, the antioxidant activity of unfermented brewer’s spent grain, which was nearly zero, increased significantly to 64 µmol/g (Trolox equivalents) and 12 mg/g (gallic acid equivalents) after SSF with *A. ibericus* [53]. Leite et al. [61] concluded that the time of SSF was a factor affecting the antioxidant activity of extracts obtained in the fermented mixture of crude olive pomace, exhausted olive pomace, and brewer’s spent grain. During the SSF of oilseed cakes (sunflower, rapeseed, and soybean), *R. oryzae*, *A. niger*, and *A. ibericus* produced lignocellulolytic enzymes, releasing phenolic compounds with antioxidant capacity. A principal component analysis demonstrated a positive correlation between the extracts with high antioxidant potential and the SSF with high enzyme production [48]. The enzymatic extract obtained after the SSF of 50% (*w*/*w*) rapeseed cake and sunflower cake with *A. niger* had an antioxidant activity higher than that of unfermented oilseed cakes [62]. SSF with a consortium of *A. niger* and *R. oryzae* proved to be a successful bioprocessing approach to improve the antioxidant activity of rapeseed cake [63]. By contrast, the SSF of a plant feedstuff mixture did not increase the total antioxidant activity, regardless of the fungi species used [55].

### 2.3. Box–Behnken Experimental Design

Considering the results obtained in the Plackett–Burman design and the data found in the literature, a Box–Behnken design was further used to find the best range of the moisture content, ammonium sulfate concentration, and CSL concentration, to maximize the protein content of fruit peels after SSF with *Aspergillus ibericus*. In general, the protein content of fruit peels increased after SSF compared to unfermented peels. The protein content varied from 7.32% (run 5) to 16.29% (run 6) in fermented OPs and from 8.35% (run 14) to 17.67% (run 2) in fermented BPs (Table 4).

In the SSF of OPs, the lowest protein content was obtained in experiment 5, with conditions of 50% moisture and 0.005 g/g ammonium sulfate. By contrast, the highest protein content was attained in experiment 6, under 70% moisture and supplemented with 0.005 g/g ammonium sulfate, which corresponded to an increase of 200% comparatively to unfermented OPs. These results suggest that high moisture content favored fungus growth and, consequently, protein synthesis, while CSL supplementation had a negative effect. Moisture content was considered a significant factor, with a positive impact on protein production (*p* = 0.003). This was confirmed by the estimated coefficient (2.31) obtained through ANOVA analysis. The lack of significance in the effects of AS and CSL may be attributed to the narrow range of concentrations studied. It is possible that the real impact of these variables was not fully observed due to the use of very low concentrations.

In the SSF of BPs, the lowest protein content was obtained in experiment 14, which was a central point for the experimental design and had conditions of 60% moisture, 0.005 g/g ammonium sulfate, and 0.005 g/g CSL. By contrast, the highest protein content was obtained with 70% moisture and 0.005 g/g CSL, which corresponded to an increase of 123% comparatively to unfermented BPs. For BPs, none of the variables were significant for protein production (*p* > 0.05) in the range of operational conditions studied. This might be due to very similar protein content within almost all experiments (an average of around 11%) (Table 4).

**Figure 2:**
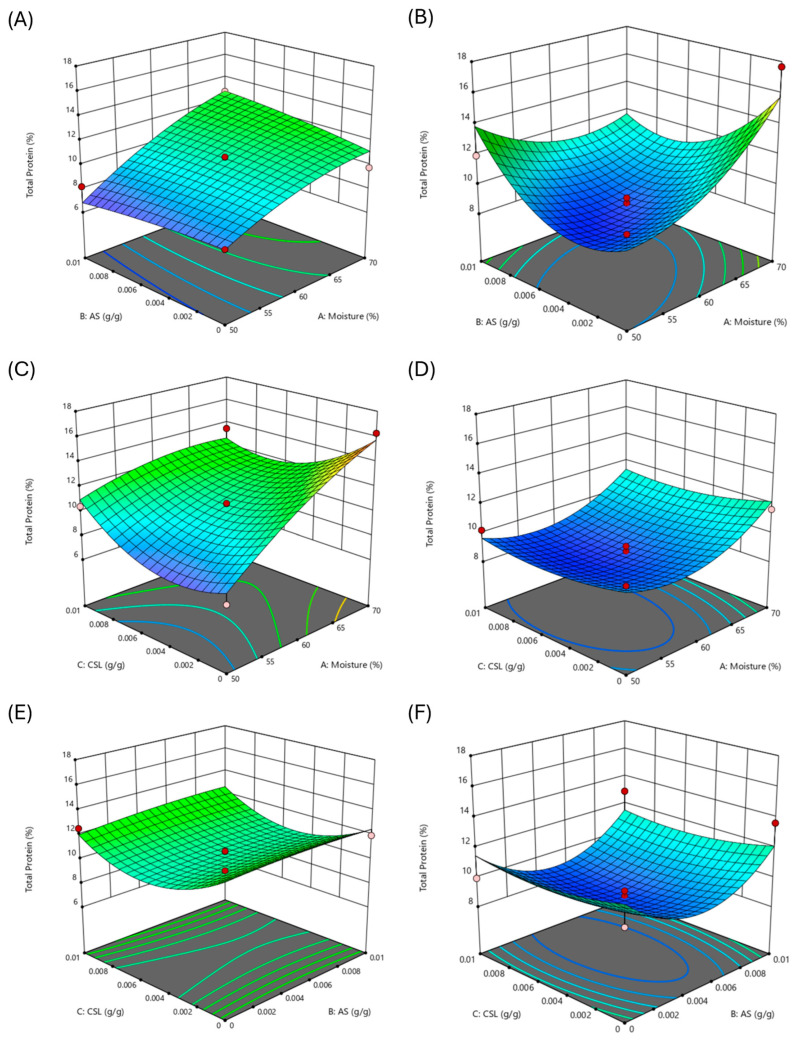
The 3D surface plots indicating the effects of moisture vs. ammonium sulfate (AS) (**A**,**B**), moisture vs. corn steep liquor (CSL) (**C**,**D**), and AS vs. CSL (**E**,**F**) on protein content obtained in solid-state fermentation of orange peels (**left column**) and banana peels (**right column**). In dark red are the design points above the predicted value, and in light red are the design points below the predicted value.

The 3D surface plots for total protein content in fermented fruit peels are depicted in Figure 2. The response surface is important for visualizing the relationship between independent variables and their responses by holding one variable at its zero level. The response surface for total protein showed that the linear polynomial equation was best fitted for all (Figure 2A–F). The response surface 3D plots show that the protein content of OPs increased by raising the initial moisture of the SSF from 50% to 70%. The effect of ammonium sulfate (AS) was not significant, and the maximum protein content can be obtained in the range of 0.004 g/g–0.01 g/g (Figure 2A). By contrast, the supplementation of OPs with corn steep liquor (CSL) had a negative effect since the highest protein content was achieved without CSL addition (Figure 2C). It can also be observed that as the initial moisture increases, the green color reduces and gradually moves to the red region, demonstrating that the total protein content is maximal with 70% moisture. The surface 3D plot of CSL vs. AS demonstrates that the maximum protein content can be obtained either at the lowest or the highest values of both supplements (Figure 2E). These findings can be related to the narrow range of AS and CSL studied and corroborate the results of the Plackett–Burman design, in which none of these factors were significant for protein production in the SSF of OPs. Thus, the maximal protein content can be attained by the SSF of OPs at 70% moisture and with (or without) the addition of AS or CSL.

The protein content in fermented BPs was improved by increasing the moisture content from 50% to 70%, as the green color of the plot reduced and gradually moved to the yellow region (Figure 2B). Still, this parameter’s effect was not as obvious as in OP experiments since at 50% moisture, there is also one peak in the graph. The interaction between CSL and moisture content revealed that moisture of 70% was significant for the maximal protein content, whereas CSL had no statistical effect since the plot is in the blue region in all ranges of CSL concentration (Figure 2D). Furthermore, no direct relation between ammonium sulfate and CSL was observed for BPs (Figure 2F). Based on these findings, the highest protein content can be attained by the SSF of BPs at 70% moisture and with (or without) the addition of AS or CSL.

In both OP and PB fermentations, the most favorable conditions for protein production were at 70% moisture, as it is a critical factor in SSF. Solids with low moisture content might cause problems with nutrient solubility from the solid substrate, ultimately reducing metabolic and enzyme activity. Additionally, it can provide poor substrate swelling and high-water tension, consequently promoting slow growth. In contrast, a high moisture content can also inhibit growth through low oxygen transfer [64]. The moisture content also proved to be a crucial factor for *A. ibericus* growth and protein improvement of brewer’s spent grain, olive pomace, exhausted grape mark, and vine-shoot trimming, and 75% moisture was shown to be the best condition [56].

Nitrogen is essential for the synthesis of proteins and nucleic acids, while minerals support a variety of structural, enzymatic, and regulatory functions. Ensuring an adequate supply of these nutrients is crucial for optimal fungal growth, development, and metabolic activity. Karmakar et al. [65] studied the addition of salts such as CaCl_2_, SrCl_2_, and NH_4_Cl in the growth of *Rhizopus oryzae* and observed an improvement in fungi biomass due to the activation of chloride channels by Ca^2+^ cations. This ability of microorganisms to use the substrates as carbon sources seems to be linked to the mineral content. Oshoma et al. [66] also observed an enhancement in *A. niger* growth and microbial protein production by supplementing BPs with minerals. Alimon et al. [67] reported that ammonium sulfate supplementation enhanced fungal biomass and protein production by delaying the sporulation of *Aspergillus niger*. Schmidt and Furlong [46] also observed a positive correlation between the increase in ammonium sulfate concentration in rice bran and *Rhizopus oryzae* biomass and protein synthesis. The results of Yasin et al. [68] showed an improvement in protein content when BPs were supplemented with 0.25% CSL in SSF with *Arachniotus ruber.* By contrast, no supplementation of BPs was needed to improve the protein content by 150% after SSF with *Phanerochaete chrysosporium* and *Candida utilis* [59].

The fermented peels with the highest protein content (run 6 for OP; run 2 for BP), as well SSF controls (non-inoculated peels) were characterized for carbohydrates, reducing sugars, crude fiber, pectin, total lipids, and antioxidant activity (Table 5).

In both control experiments (OPs and BPs without inoculation), the content of carbohydrates, reducing sugars, total protein, crude fiber, pectin, and total lipids was similar to those of natural peels (Table 1), demonstrating that the differences between natural and fermented peels were due to the fermentation with *A. ibericus*. The composition of macrominerals of non-inoculated peels was similar to that of natural peels, but an increase in macromineral concentration was attained after SSF owing to the inherent composition of fungal biomass, which is also composed by these components.

Solid-state fermentation changed the nutritional profile of orange peels (OPs) and banana peels (BPs), resulting in a composition distinct from both the natural peels and the control experiments (non-inoculated peels). Specifically, the total protein content increased by 167% and 111% in fermented OPs and BPs, respectively, compared to unfermented peels. The consumption of protein has been recognized as indispensable for maintaining a healthy diet and lifestyle. Benefits include tissue development and repair, enzyme and hormone production, and immune system, energy supply, and muscle mass maintenance [69]. The reduction of carbohydrates and soluble sugars, which are commonly associated with a high caloric content in food, is beneficial for individuals with diabetes or insulin resistance since lower sugar intake can lead to more stable blood sugar levels. Fermented fruit peels are rich in dietary fibers, particularly BPs, which is advantageous for digestive health, blood sugar, cholesterol, weight control, and a lower risk of chronic diseases [70]. Furthermore, their mineral content can improve bone health, muscle and neuron function, metabolism, and energy production [71]. The lipid content of fermented peels increased compared to unfermented peels due to the lipidic composition of the fungi membranes and lipids accumulated intracellularly, which in some cases are rich in mono- and poly-unsaturated fatty acids [72]. The SSF of OPs and BPs by *A. ibericus* also demonstrated an improvement in their antioxidant properties, which have the potential to be used for the production of functional foods, contributing to the reduction in chronic diseases (e.g., heart disease, diabetes, and cancer) [71].

## 3. Materials and Methods

### 3.1. Raw Materials

Orange (OPs) and banana (BPs) peels, collected from a public school canteen, were washed, ground in a food processor (particle size 0.5–1 cm), dried at 60 °C in a ventilated oven, and stored at −20 °C until use. OPs and BPs were characterized for initial moisture, total protein, total lipids, total carbohydrates, soluble reducing sugars, pectin, crude fiber, total soluble phenols, ashes, antioxidant activity, and mineral composition.

### 3.2. Microorganisms

*Aspergillus ibericus* MUM 03.49 (does not produce mycotoxins) and *Rhizopus oryzae* MUM 10.260 (GRAS status) were obtained from the culture collection of Micoteca of Universidade do Minho (MUM, Braga, Portugal). The fungal strains grew in potato dextrose agar (PDA) (4 g/L potato extract, 20 g/L dextrose, 15 g/L agar) at 25 °C for 5 days and were stored at 4 °C for a maximum of 2 weeks.

### 3.3. Solid-State Fermentation (SSF) of Fruit Peels (FPs)

SSF experiments were carried out in 250 mL Erlenmeyer flasks containing 5 g of dry peels, at 25 °C in a ventilated oven without agitation. The spore suspension of each fungal strain was prepared by adding a sterile peptone solution (1 g/L peptone and 0.1 g/L Tween 80) to the 5-day-grown agar plates. After appropriate dilution, 1 × 10^7^ spores/g solid or 5 × 10^7^ spores/g solid suspensions were used to inoculate fruit peels. The initial moisture content, spores’ concentration, fermentation time, fungal species, and ammonium sulfate and corn steep liquor (CSL) concentrations were adjusted according to the experimental assay matrixes (Plackett–Burman and Box–Behnken). Control experiments without inoculation for each experimental run were also performed.

After SSF, the fermented FPs were mixed to homogenize all components, and 2 g was sampled to quantify total protein, moisture, carbohydrates, crude fiber, pectin, and lipids. Soluble reducing sugars and antioxidant activity were measured in liquid extracts obtained by extraction of fermented FPs with distilled water (1:10 *w*/*v*), in an orbital incubator at 200 rpm for 30 min (room temperature). The liquid extracts were then filtered with Whatman^®^ qualitative filter paper.

#### 3.3.1. Plackett–Burman Design (PBD)

A Plackett–Burman design was used to study the main effects of six independent factors (fermentation time, inoculum size, moisture, ammonium sulfate concentration, CSL concentration, and fungal species) on total protein, soluble reducing sugars, and antioxidant activity of fermented FP. These factors were tested at two levels for a total of twelve experiments (Table 6), each one carried out once. The Plackett–Burman design experimental data were adjusted to a linear model, that establishes a relationship between response variables (*Y*), factors (*x_i_*), and their main effect coefficients (*a_i_*):

(1) $$Y=A+\sum_{i}a_{i}\times x_{i}$$

#### 3.3.2. Box–Behnken Experimental Design

The Box–Behnken design was used for optimization of the selected SSF factors (moisture, ammonium sulfate concentration, and CSL concentration) to maximize the total protein content of fermented OPs and BPs (Table 7). A total of 15 runs, 3 of which were central points, were carried out once. In the final experiments (verification of maximum conditions predicted by Box–Behnken design), the fermented peels were also characterized for moisture, carbohydrates, reducing sugars, crude fiber, pectin, lipid content, and antioxidant activity.

The Box–Behnken design experimental data were adjusted to a quadratic model, that established a relationship between response variables (*Y*), factors (*x_i_*), main effect coefficients (*a_i_*), and interaction effects coefficients (*a_ij_*):

(2) $$Y=A+\sum_{i}a_{i}\times x_{i}+\sum_{i}a_{ii}\times x_{i}^{2}+\sum_{ij}a_{ij}\times x_{i}\times x_{j}$$

### 3.4. Analytical Methods

Moisture and ashes were quantified by AOAC methods [73]. The total protein content was assessed by the Kjeldahl method. The samples were digested with H_2_SO_4_ 96% for 1 h at 420 °C, neutralized with NaOH 40%, and analyzed for total nitrogen content after distillation with boric acid. Following the nitrogen quantification, crude protein was estimated using a conversion factor of 6.25. The total lipids were quantified by the Soxhlet method using petroleum ether as a solvent at 70 °C. Total carbohydrates were measured after acidic hydrolysis of fruit peels. Briefly, 10 mL of H_2_SO_4_ 1.5 M was added to the solid sample (0.1 g–0.2 g) and incubated in a water bath at 100 °C for 20 min. After cooling, 12 mL of NaOH 10% was added to each tube, and the mixture was filtered and adjusted to 100 mL (in a volumetric flask) with distilled water. The reducing sugars were quantified by the 3,5-Dinitrosalicylic acid (DNS) method, using glucose as the standard. The content of total carbohydrates was quantified by the following:

(3) $$HC\;\left(\%\right)=\frac{C_{glucose}}{m_{sample}}\times 10$$

where *C_glucose_* is the concentration of glucose determined by the DNS method and *m_sample_* is the mass of the sample used in the procedure. Pectin was quantified according to Rodsamran and Sothornvit [74] with some modifications: HCl 0.05 M (1:20 *v*/*w*) was added to the dried fruit peels (10 g) and the mixture was agitated in a water bath for 1 h at 95 °C; after the filtration, the remaining solid was subjected to another extraction under the same conditions. Absolute ethanol (1:2) was added to the filtrates and agitated in an orbital incubator for 10 min at 200 rpm (room temperature) and stored for 2 h in static conditions. The precipitated pectin was vacuum-filtered, washed with absolute ethanol and acetone (3:1), and dried at 40 °C overnight before weighing.

The crude fiber was quantified by the AOAC Official Method 962.09 [73]. The mineral elements were measured by Inductively Coupled Plasma Optical Emission Spectrometry (ICP-OES, PerkinElmer, Inc., Waltham, MA, USA). Fruit peels were previously digested using a defined protocol (fresh fruit protocol) from the Berghof microwave digestion system (Speedwave four DAP-60+, Berghof Products + Instruments GmbH, Eningen, Germany). The digested liquid samples were analyzed in the ICP-OES equipment (Optima 8000, PerkinElmer, Waltham, MA, USA) under the following operating conditions: radio frequency power of 1400 W; 12 L/min argon plasma flow; 0.2 L/min auxiliary gas flow; and 0.75 L/min nebulizer gas flow. The plasma view was axial for all analyzed elements. The wavelengths (nm) used for each element were as follows: Cu, 324.752; K, 766.490; Mg, 280.271; Na, 588.995; P, 213.617; Ca, 317.933; Fe, 238.204; Mn, 259.372; Ni, 221.648; Zn, 213.857; Sr, 407.771; and Ba, 493.408.

Soluble reducing sugars, total phenols, and antioxidant activity were quantified in the liquid extracts of unfermented and fermented peels. The soluble reducing sugars were measured through the 3,5-Dinitrosalicylic acid (DNS) method using glucose as the standard. Total phenols were determined using the Folin–Ciocalteu method (Commission Regulation (EEC) No. 2676/90). Antioxidant activity was quantified by a 2,2-diphenyl-1-picrylhydrazyl (DPPH) radical scavenging assay as described by Dulf [75] and adapted by Estevão-Rodrigues et al. [53]. The calibration curve was prepared using 6-hydroxy-2,5,7,8-tetramethyl chroman-2-carboxylic acid (Trolox) as the standard. The results were expressed in µmol of Trolox equivalents (TEs) per gram of dry solid substrate (µmol/g).

### 3.5. Statistical Analysis

The data of the Plackett–Burman experimental design were analyzed in the Statgraphics Centurion 19.05.01 program, and the data of the Box–Behnken experimental design were analyzed in the Design Expert 13.0.5.0 program. Statistical significance was considered with a confidence interval of 95%. One-way analysis of variance (ANOVA) was performed, and Student´s *t*-test was used to detect significant differences among the means (*p* < 0.05) of fruit peels’ composition (Table 1, Table 2 and Table 5).

## 4. Conclusions

This study demonstrated the potential of solid-state fermentation (SSF) to improve the nutritional value of orange and banana peels, particularly using the fungus *A. ibericus*. The fermented peels were enriched in fiber and minerals, and the total protein and total lipids were considerably higher than those of unfermented peels. Particularly, total protein increased by 200% in orange peels (OPs) and 123% in banana peels (BPs). Furthermore, an improvement in antioxidant activity after SSF was also observed for both fruit peels. By contrast, the content of carbohydrates and soluble sugars in fermented fruit peels was significantly lower than that of unfermented peels. According to these findings, an enhancement in the nutritional value of the fruit peels may be effectively achieved using SSF, proving the potential to be used as food due to its high nutritious content. This approach could offer a sustainable solution, not only by upcycling food byproducts but also by potentially relieving pressure on traditional food production systems.

## Figures and Tables

**Table 1:** Composition of orange and banana peels. Data (% *w*/*w* in dry basis) are the average ± standard deviation of 3 analyses. Values followed by the same letter in each row do not present statistically significant differences (*p* ≥ 0.05).

Component (%)	Orange Peels	Banana Peels
Moisture	82.04 ± 0.53 ^a^	91.62 ± 0.81 ^b^
Carbohydrates	59.15 ± 5.61 ^a^	35.63 ± 2.64 ^b^
Reducing sugars *	26.69 ± 2.14 ^a^	15.46 ± 1.35 ^b^
Crude fiber	11.81 ± 1.05 ^a^	21.33 ± 3.55 ^b^
Pectin	19.26 ± 0.80 ^a^	4.11 ± 0.62 ^b^
Total protein	5.44 ± 0.93 ^a^	7.92 ± 0.53 ^b^
Total lipids	1.70 ± 0.04 ^a^	5.79 ± 2.47 ^b^
Ashes	3.09 ± 0.80 ^a^	13.52 ± 1.39 ^b^
Total phenols (mg/g) *	9.33 ± 1.75 ^a^	2.46 ± 1.20 ^b^
Antioxidant activity (μmol/g) *	7.33 ± 2.00 ^a^	4.17 ± 1.78 ^a^

* Measured in aqueous extract.

**Table 2:** Characterization of mineral elements of orange and banana peels. Data (on a dry basis) are the average ± standard deviation of 2 independent analyses. Values followed by the same letter in each row do not present statistically significant differences (*p* ≥ 0.05).

Elements	Orange Peels	Banana Peels
Macrominerals (mg/g)		
K	1.15 ± 0.04 ^a^	4.50 ± 0.47 ^b^
Ca	1.19 ± 0.03 ^a^	0.52 ± 0.04 ^b^
Na	0.04 ± 0.01 ^a^	0.041 ± 0.001 ^a^
P	0.14 ± 0.04 ^a^	0.252 ± 0.002 ^a^
Mg	0.16 ± 0.03 ^a^	0.20 ± 0.02 ^a^
Microminerals (μg/g)		
Fe	1.12 ± 0.59 ^a^	2.39 ± 0.59 ^a^
Cu	1.87 ± 0.47 ^a^	0.96 ± 0.11 ^a^
Zn	2.15 ± 0.61 ^a^	2.81 ± 0.24 ^a^
Mn	0.82 ± 0.03 ^a^	6.57 ± 0.91 ^b^
Sr	7.92 ± 0.38 ^a^	4.51 ± 0.44 ^b^
Ni	0.11 ± 0.01 ^a^	0.11 ± 0.03 ^a^
Br	1.08 ± 0.03 ^a^	1.68 ± 0.08 ^b^

**Table 3:** Experimental results of total protein (TP), reducing sugars (RSs), and antioxidant activity (AA) obtained in Plackett–Burman variable screening analysis after solid-state fermentation of orange and banana peels. The Plackett–Burman matrix, comprising 12 runs, was used to screen the main effects of six factors at 2 levels—fermentation time (7 or 14 days), inoculum size (1 × 10^7^ or 5 × 10^7^ spores/g), moisture content (60% or 75%), ammonium sulfate (AS) concentration (0 or 0.01 g/g), corn steep liquor (CSL) concentration (0 or 0.01 g/g), and fungal species (*A. ibericus* or *R. oryzae*. Data are represented in *w*/*w* (dry basis).

	Orange Peels			Banana Peels		
Run	TP (%)	RS (%)	AA (μmol/g)	TP (%)	RS (%)	AA (μmol/g)
1	6.30	15.54	15.56	9.21	8.44	9.36
2	10.50	2.71	15.08	9.01	5.31	9.37
3	10.07	22.34	14.02	9.44	3.60	10.14
4	6.60	32.92	13.60	11.01	2.22	7.36
5	6.02	10.34	13.43	8.39	2.66	7.33
6	7.92	15.68	13.52	7.95	14.99	6.30
7	13.33	36.44	14.00	9.39	2.44	8.23
8	5.64	20.07	14.55	8.24	11.00	8.07
9	10.73	37.34	16.12	9.05	12.81	5.22
10	7.07	9.02	15.92	9.04	13.39	12.89
11	12.28	29.97	13.81	8.45	11.67	12.93
12	5.44	21.92	14.53	7.74	12.26	14.66

**Table 4:** Experimental results of total protein content in fermented orange peels (OPs) and in fermented banana peels (BPs) obtained for Box–Behnken optimization analysis. The Box–Behnken matrix, comprising 15 runs with 3 central points, was used to optimize the level of moisture (50%, 60%, 70%), ammonium sulfate (AS) concentration (0, 0.005 g/g, 0.01 g/g), and corn steep liquor (CSL) concentration (0, 0.005 g/g, 0.01 g/g). Data are represented in *w*/*w* (dry basis).

Run	Moisture	AS	CSL	Total Protein (%)	
				OP	BP
1	−1	−1	0	7.89	10.67
2	1	−1	0	9.78	17.67
3	−1	1	0	8.18	11.92
4	1	1	0	12.74	10.75
5	−1	0	−1	7.32	10.39
6	1	0	−1	16.29	11.63
7	−1	0	1	10.41	10.19
8	1	0	1	13.49	10.96
9	0	−1	−1	13.31	10.65
10	0	1	−1	11.96	13.65
11	0	−1	1	12.53	9.94
12	0	1	1	11.78	12.92
13	0	0	0	10.32	8.72
14	0	0	0	10.64	8.35
15	0	0	0	10.68	9.09

**Table 5:** Composition of fermented orange and banana peels and SSF controls obtained after solid-state fermentation at 25 °C for 7 days with *Aspergillus ibericus*. Data (*w*/*w* in dry basis) are presented as the average ± standard deviation of 2 independent analyses. For each fruit peel, values followed by the same letter in each row do not present statistically significant differences (*p* ≥ 0.05).

	Orange Peels		Banana Peels	
Component	Control	Fermented	Control	Fermented
Moisture (%)	79.24 ± 0.65 ^a^	80.65 ± 0.71 ^a^	86.16 ± 0.54 ^a^	87.54 ± 0.21 ^a^
Carbohydrates (%)	58.63 ± 3.91 ^a^	11.98 ± 1.18 ^b^	32.22 ± 2.40 ^a^	16.91 ± 1.87 ^b^
Reducing sugars (%)	24.34 ± 3.02 ^a^	1.72 ± 0.17 ^b^	12.91 ± 2.92 ^a^	4.03 ± 0.14 ^b^
Crude fiber (%)	10.92 ± 1.24 ^a^	9.86 ± 1.94 ^a^	22.48 ± 3.69 ^a^	23.88 ± 4.12 ^a^
Pectin (%)	20.88 ± 2.07 ^a^	3.81 ± 1.10 ^b^	4.28 ± 0.14 ^a^	3.76 ± 0.27 ^a^
Total protein (%)	5.96 ± 0.70 ^a^	16.23 ± 0.43 ^b^	8.28 ± 0.45 ^a^	17.49 ± 0.78 ^b^
Total lipids (%)	1.67 ± 0.03 ^a^	4.22 ± 0.11 ^b^	5.35 ± 0.87 ^a^	9.41 ± 0.60 ^b^
K (mg/g)	1.11 ± 0.11 ^a^	1.97 ± 0.17 ^b^	3.19 ± 0.87 ^a^	4.82 ± 0.68 ^a^
Ca (mg/g)	1.16 ± 0.14 ^a^	1.88 ± 0.46 ^a^	0.48 ± 0.16 ^a^	0.81 ± 0.41 ^a^
Na (mg/g)	0.046 ± 0.004 ^a^	0.08 ± 0.02 ^a^	0.04 ± 0.01 ^a^	0.05 ± 0.01 ^a^
P (mg/g)	0.14 ± 0.01 ^a^	0.24 ± 0.01 ^b^	0.25 ± 0.01 ^a^	0.38 ± 0.15 ^a^
Mg (mg/g)	0.12 ± 0.02 ^a^	0.18 ± 0.02 ^a^	0.19 ± 0.02 ^a^	0.20 ± 0.04 ^a^
Antioxidant activity (μmol/g)	9.79 ± 1.49 ^a^	20.02 ± 1.26 ^b^	5.91 ± 1.01 ^a^	9.75 ± 0.56 ^b^

**Table 6:** Plackett–Burman design including six factors and minimum (−1) and maximum (+1) coded values used to study the main effects of fermentation time, inoculum size, moisture content, ammonium sulfate (AS) concentration, corn steep liquor (CSL) concentration, and fungal species, at 2 levels.

Run	Time (days)	Inoculum Size (Spores/g)	Moisture (%)	AS (g/g)	CSL (g/g)	Fungal Species
1	−1	1	1	1	−1	1
2	−1	1	1	−1	1	−1
3	1	−1	1	−1	−1	−1
4	1	−1	1	1	−1	1
5	1	1	1	−1	1	1
6	−1	−1	−1	−1	−1	−1
7	−1	−1	1	1	1	−1
8	1	−1	−1	−1	1	1
9	1	1	−1	1	−1	−1
10	−1	−1	−1	1	1	1
11	1	1	−1	1	1	−1
12	−1	1	−1	−1	−1	1
−1	7	1 × 10^7^	60	0	0	*Aspergillus ibericus*
1	14	5 × 10^7^	75	0.01	0.01	*Rhizopus oryzae*

**Table 7:** Levels of independent variables studied in the Box–Behnken design.

Variables	−1	0	+1
Moisture (%)	50	60	70
AS concentration (g/g)	0	0.005	0.01
CSL concentration (g/g)	0	0.005	0.01

## Data Availability

Data is contained within the article.
